# Fournier's Gangrene as a Postoperative Complication of Inguinal Hernia Repair

**DOI:** 10.1155/2014/408217

**Published:** 2014-11-19

**Authors:** Tolga Dinc, Selami Ilgaz Kayilioglu, Isa Sozen, Baris Dogu Yildiz, Faruk Coskun

**Affiliations:** ^1^Department of General Surgery, Ankara Numune Training and Research Hospital, Altindag, 06100 Ankara, Turkey; ^2^Department of General Surgery, Corum Training and Research Hospital, Hitit University School of Medicine, Turkey

## Abstract

Fournier's gangrene is the necrotizing fasciitis of perianal, genitourinary, and perineal regions. Herein, we present a case of scrotal Fournier's gangrene as a postoperative complication of inguinal hernia repair. A 51-year-old male with giant indirect hernia is presented. Patient underwent inguinal hernia repair, and after an unproblematic recovery period, he was discharged. He applied to our outpatient clinic on the fifth day with swollen and painful scrotum and it turned out to be Fournier's gangrene. Polypropylene mesh was not infected. Patient recovered and was discharged after repeated debridements. Basic principles in treatment of Fournier's gangrene are comprised of initial resuscitation, broad-spectrum antibiotics therapy, and early aggressive debridement. In the management of presented case, aggressive debridement was made right after diagnosis and broad-spectrum antibiotics were given to the hemodynamically stable patient. In these circumstances, the important question is whether we could prevent occurrence of Fournier's gangrene.

## 1. Introduction 

Fournier's gangrene is the necrotizing fasciitis of perianal, genitourinary, and perineal regions [1, 2]. Multiple etiological factors are responsible. Fournier's gangrene of scrotum is mostly caused by previous scrotum surgery and local infections [3, 4]. Gangrene may expand to abdominal wall, intra-abdominal structures, and even retroperitoneal tissues. Despite improvements in practice of initial resuscitation, antibiotics, and anesthesia, mortality rate of Fournier's gangrene can be around 75% [2, 5]. Herein, we present a case of scrotal Fournier's gangrene as a postoperative complication of inguinal hernia repair.

## 2. Case Report

**Figure 1:**
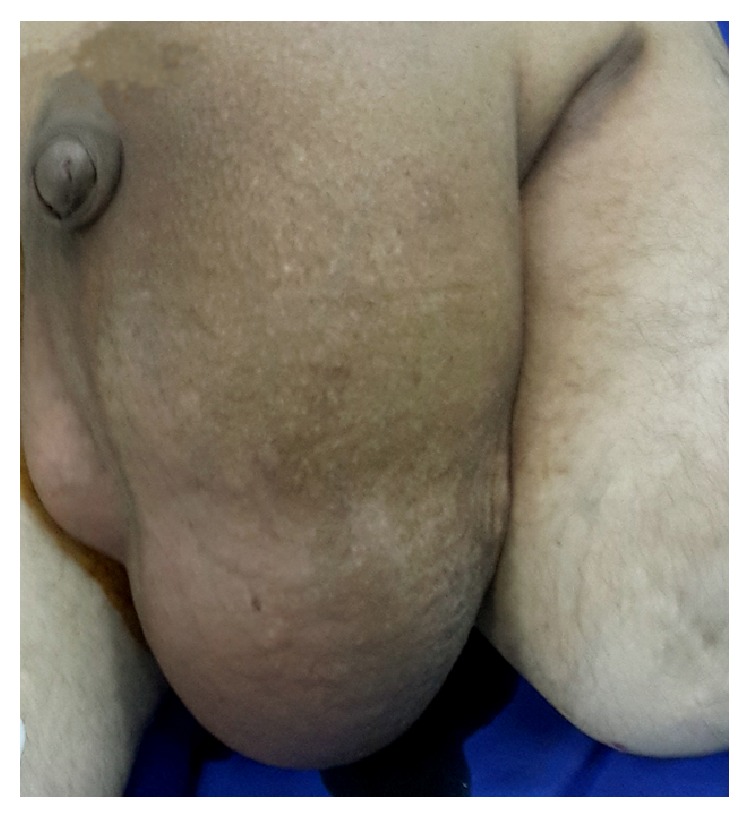
Preoperative view of left scrotal inguinal hernia.

**Figure 2:**
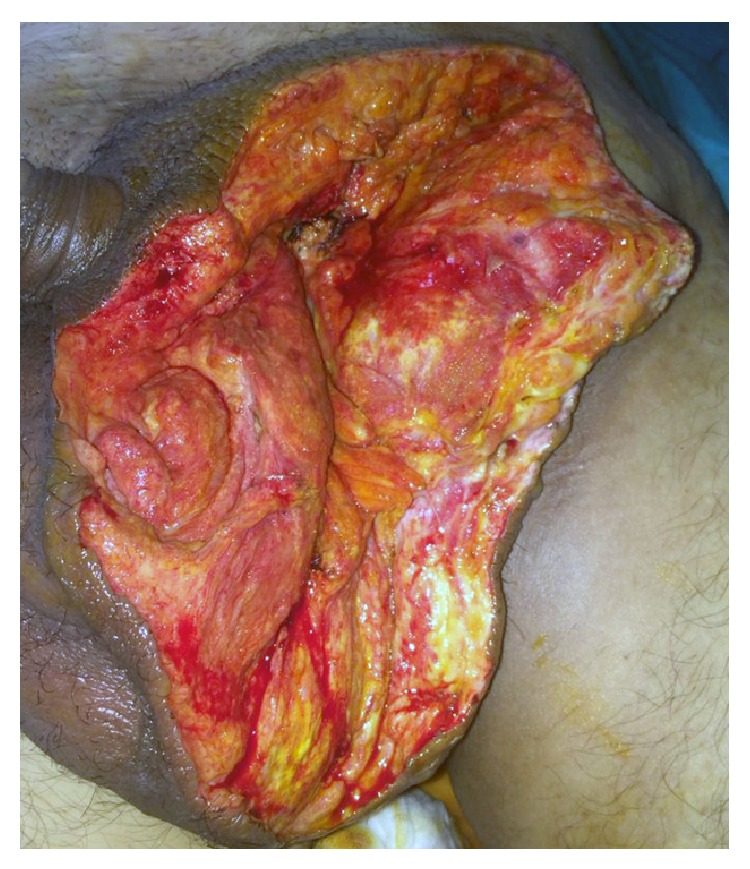
View of the surgical site after orchiectomy and debridement.

**Figure 3:**
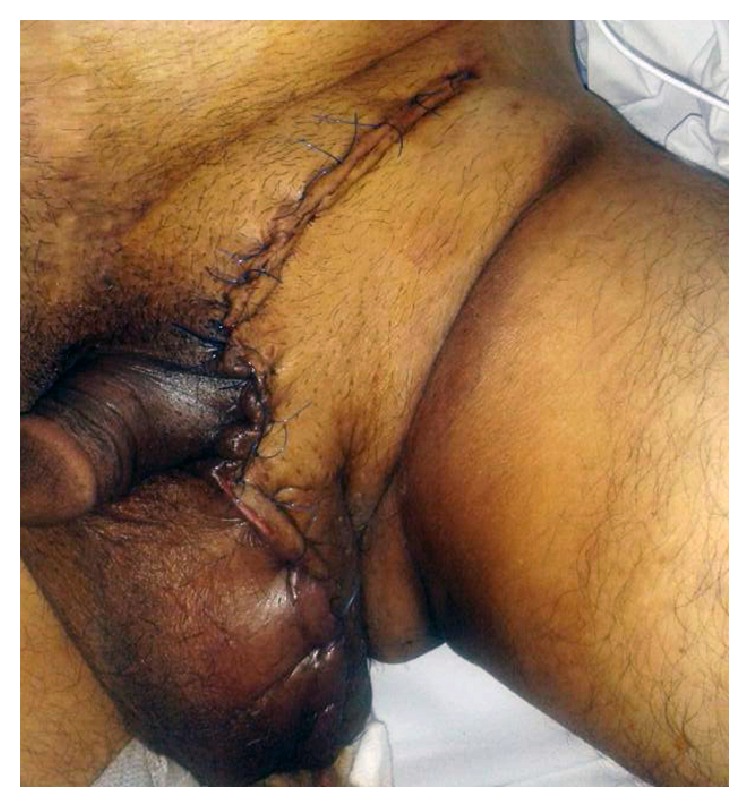
Skin is totally sutured and closed after multiple debridement sessions.

A 51-year-old male, suffering from left inguinal hernia, appealed to our institution. His hernia had an 18-year-old history according to patient's statement (Figure 1). His medical history included ten pack-years of smoking, well-controlled diabetes, and above knee amputation due to trauma (15 years ago). Physical examination revealed left indirect scrotal inguinal hernia. Patient is taken to the operating room with minor predicted operative risk. Following antimicrobial prophylaxis with a second generation broad-spectrum cephalosporin, left inguinal oblique incision was made and hernia sac was explored. Caecum, appendix, descending colon, sigmoid colon, and small intestines were found in indirect hernia sac. No sign of any strangulation was present. Hernia sac was dissected, all herniated organs were reduced into abdomen, and hernia sac was totally removed. We placed a polypropylene mesh and fixated it. A suction drainage tube which was lying near spermatic cord going into scrotum was placed. Patient was carefully monitored in case of abdominal compartment syndrome. On the postoperative tenth day, the amount of drainage was below 10 milliliters a day and no postoperative complications were observed. Patient was discharged. On the fifth day after discharge, patient applied to our outpatient clinic, suffering from scrotal pain and swelling. Scrotum was swollen, tense, and tender to the touch. Inadequate hygiene was noticed. Emergency surgery was decided as the method of lowering intrascrotal pressure and diagnosing. After vertical scrotal incision, large amount of cloudy serous fluid was drained. Left testicle and surrounding soft tissue were significantly necrotic. There was no sign of infection or necrosis in the soft tissue surrounding the previously placed polypropylene graft. Orchiectomy and debridement of necrotic soft tissue were performed (Figure 2). Postoperative care included antimicrobial therapy (for starters empiric broad-spectrum antibiotics and then agent-specific imipenem, daptomycin, and ciprofloxacin) and daily debridement and dressings. Wound was partially sutured step by step in debridement sessions (Figure 3). Systemic and local infection findings disappeared in days. Patient was discharged after 16 days of hospital stay.

## 3. Discussion

Fournier's gangrene is defined as aggressive necrotizing fasciitis of external genitalia and perineum [1]. Urogenital tract, anorectal lesions, or skin itself is commonly the resource and entrance of the infectious agents [6]. Diabetes, chronic alcohol use, steroid intake, malignancies, anticancer agents, HIV infection, and neurologic deficits that impair physical activities are risk factors for Fournier's gangrene. In this particular case, diabetes, chronic use of alcohol, and physical impairment due to the above knee amputation was present. Besides these, we think that patient's inadequate hygiene was also an effective factor.

Infectious complications rates following hernia repair surgery are reported around 1–4% in the literature [4]. As most of these complications are wound infections, mesh removal becomes necessary in only small portion of these cases. Fournier's gangrene following hernia repair surgery is an extremely rare condition. There is limited number of papers that reported Fournier's gangrene as a result of untreated complicated inguinal hernias, like Richter's hernia [7], incarcerated hernia [8], giant neglected bilateral hernia [9], or organ perforation [10]. As far as we know, this is the very first report of a Fournier's gangrene as a postoperative complication of inguinal hernia repair surgery.

In our case, Lichtenstein repair was performed due to giant indirect inguinal hernia. As a part of the procedure, after reduction of hernia contents, hernia sac was removed totally. Although a drainage tube was placed and monitored for ten days and removed when drainage was very low, after removal of tube, we think that fluid sequestration continued and this led to severe increase in intrascrotal pressure. Probably, increased pressure impaired blood supply of testicle and surrounding soft tissue, and infection supervened.

Basic principles in treatment of Fournier's gangrene are comprised of initial resuscitation, broad-spectrum antibiotics therapy, and early aggressive debridement [1]. In the management of presented case, aggressive debridement was made right after diagnosis and broad-spectrum antibiotics were given to the hemodynamically stable patient. In these circumstances, the important question is whether we could prevent occurrence of Fournier's gangrene. Traditionally, drain tube usage and longer hospital stay times are preferred more commonly in scrotal hernia patients. In this case, even the length of hospital stay was quite long and suction drainage tube was used and monitored for enough time; Fournier's gangrene occurred on the fifth day after discharge. According to this, for giant scrotal hernias, as in our case, standard postoperative care might prove insufficient.

Moreover, postoperative hygienic status of our patient probably played a role in occurrence of Fournier's gangrene. In our opinion, genital hygiene in perioperative period is commonly overlooked by surgeons.
